# Observations on Extraction of Diseased Ovaria; Illustrated

**Published:** 1825-10-01

**Authors:** 


*Observations on Extraction of Diseased Ovaria; illustrated by Plates colored after Nature.* By John Lizars, Surgeon, author of the System of Anatomical Plates, &c. Folio, pp. 24, five coloured plates. Edinburgh, May, 1825.

We have been false prophets—not the first, however, in the world. We expressed our disbelief of the successful removal of diseased ovaria; and this disbelief was founded on what we had seen in the examination of bodies—and what we had read of the operations themselves. When a diseased ovarium arrives at that size which requires an operation, we think there must be such adhesions to the neighbouring parts, as to render success, after an operation, next to a miracle. In respect to the recorded operations, it is to be observed that, previously to Mr. Lizars' case, none have been performed in this country—for the operation of Mr. Pott, as will be presently shewn, deserves not to be ranked as an extraction of ovarium from the abdomen. As to the operations said to have been performed in Kentucky, by Dr. Macdowal, we are as sceptical as ever. The details themselves bear intrinsic marks of Munchausenism. But further. How is it, we ask, that these cases were not published in America—and that they were only found eight or nine years afterwards among the posthumous papers of Mr. John Bell? Our American brethren are, we can assure Mr. Lizars, not at all behind hand in publishing their operations; nor is there any lack of vehicles for conveying exploits, like those under consideration, to the world at large. We regard the utter silence, therefore, of the operator, as a strong confirmation of our doubts respecting the authenticity of the operations.*

There can be no doubt, however, that the extraction of a diseased ovarium has now been performed with success in this country; and we hasten to communicate the information to our readers.

Various modes of treatment, Mr. Lizars observes, have been adopted for the cure of ovarian disease, of which there is great variety. Puncturing, and keeping open the orifice, so as to seize hold of the sac and remove it gradually, has effected a permanent cure—so, also, has the introduction of a seton. Lastly; gastrotomy and the entire removal of the sac has, it is said, been successful. L'Aumonier, chief surgeon of the great hospital at Rouen, is said to have extirpated the ovarium successfully, about 50 years ago; and some similar operations are recorded in Germany and France. "Mr. Pott extirpated first the one ovarium and then the other; but these appeared as tumours in the groins, resembling herniæ, *so that they did not require to lay open the abdomen.*" We pass over Dr. Macdowal's cases, as we have already commented on them in our number than 20 years ago—and that it has since been recorded in the sister country by Dr. Mills.—*Rev.*

* See Vol. II. of this series, p. 216.

for January, 1825, p. 216, and also Mr. Lizars' own case, where no tumour was found on laying open the abdomen. We, therefore, come to the main facts of the present publication.

*Case* 1. Janet J———, aged 36 years, unmarried, of sallow complexion, and possessing considerable muscular strength, was affected with an abdominal enlargement, resembling that of a woman in the eighth month of pregnancy. On examination, the abdomen appeared distended with a fluid and a large hard tumour, like a pregnant uterus, or extra-uterine conception. In one place the tumour was round, like the head of a fœtus, while, in another place, there was something to be felt like an elbow or knee-joint. "*The whole mass could be rolled from side to side, floating in a fluid.*" On examination, per vaginam, the os uteri could be distinctly felt, beyond which a tumour, like the head of a fœtus, and immoveable. Per rectum, the tumour is still more distinctly felt. The patient complains of pains in the lumbar and sacral regions, with considerable oppression of breathing. Her own history is as follows:—about six years ago, after exposure to cold, she was seized with severe pain in the back and violent vomiting, which continued for nearly six days. At this time she perceived a tumour in the right iliac region, about the size of a fist—at first immoveable, but, as it progressively enlarged, becoming more and more mobile. Her catamenia had been pretty regular, but scanty—bowels constipated. She has undergone two courses of mercury, and repeated courses of diuretics, without benefit. Of all the latter medicines, the supertartrate of potass has had the greatest effect in removing the ascites—but the tumour then only appeared more evident.

This poor woman, becoming miserable herself, and a burthen to her relations, implored an operation, to which Mr. Lizars at length yielded. This heroic procedure we shall record in the words of the operator.

"The day before the operation, she had a smart dose of the compound powder of jalap, which operated briskly. This day, Sunday, the 27th of February, 1825, at 1 o'clock, P. M. I began the operation, the patient being placed on her back, on a table covered with blankets, and the temperature of the room heated to 75° of Fahrenheit. The external incision having extended through the skin and adipose substance, from the ensiform cartilage to the symphysis pubis, a little on the left of the linea alba, I cautiously cut through the muscles and the peritoneum, near the umbilicus, making a small aperture into the latter, when the serous fluid flowed freely out, which was also collected by means of saucers and sponges: as the fluid ceased to flow, the wound was enlarged downwards. The whole measured about a gallon and a half. The wound was then enlarged upwards to the sternum, making that in the peritoneum correspond with the wound in the integuments; when the tumour appeared occupying the greater portion of the abdomen, and resembled the uterus in the eighth or ninth month of gestation, as represented in Plate I. I now laid hold of the tumour, brought it beyond the parietes of the abdomen, and gave it into the hands of my assistant, Mr. Macrae, as its weight threatened to drag the uterus; I then passed my fingers around the pedicle, which appeared the broad ligament of the uterus, soft, flaccid, and healthy, and about an inch and a half in length, the fundus uteri being elevated about an inch above, or atlantad to, the crista pubis. A ligature, composed of two strong threads, waxed, was next passed round this pedicle, and tied intermediate between the fundus uteri and the tumour, transfixing the pedicle between the noose of the ligature and the tumour, to prevent the noose slipping off. Lastly, I cut across the pedicle close to the tumour. During the progress of the operation, she complained of pain in the lumbar and sacral regions, which appeared to arise from the dragging of the tumour—a circumstance scarcely possible to be avoided, with all our care. My next object was to ascertain the condition of the uterus, as I was prepared to remove it had it been diseased; the uterus, however, was perfectly soft, and only a little enlarged. The other ovary was increased to nearly the size of the fourth part of the one removed, and was adhering on the right side to the parietes of the pelvis, and to the uterus, but comparatively free on the left side. While examining this, the gentlemen around me begged me to desist, in which I concurred, conceiving, that as the uterus was elevated above the brim of the pelvis, and the ovary not tied down by adhesions to the bottom of the pelvis, there might be hopes of its rising after the other had been detached, and that it might be extirpated afterwards. I now proceeded to bring the edges of the wound in apposition, by the employment of ligatures and adhesive straps; of the former there were seven, and of the latter nine, the wound itself being twelve inches. I regret that I employed so few ligatures, for, in wounds of the abdomen, they are particularly useful, to prevent any protrusion of the viscera, and to give support in all the motions of the abdomen during the cure. When the ligatures and adhesive straps had been applied, and the ligature encircling the pedicle carefully let out, compresses of lint and linen were put on, and the abdomen encircled with a shawl, as a binder employed after accouchement. This I found, in the former case, much more serviceable than a nine or twenty-tailed bandage, or stays, or any other fashionable dress. The thermometer in the room had by this time risen to 80°. I now laid my patient carefully in bed on her back, and considering that I had, to my own, as well as the satisfaction of all present, taken precautions against every risk of hæmorrhage, I left her to the care of her sister-in-law, with strict injunctions to keep her quiet, and to let her have only plain or toast-water. Apprehensive, therefore, only of inflammation, I requested an intelligent pupil, Mr. Milroy, surgeon, to call every two hours, and, on the least appearance of a rise in the pulse, or increase of pain, to abstract blood to fainting.

"The operation was finished about one o'clock; my pupil, Mr. Milroy, called between two and three o'clock, and found her sleeping, and doing well; I called myself within a few minutes to four o'clock, and met one of her relations on his way for me, as she was dying from loss of blood. I found the binder, dressings, and a considerable space of the sheets, drenched with blood, my patient scarcely able to articulate, her voice hardly audible, her face cold, and covered with clammy sweat, and the hands and feet equally cold, with no pulse at the wrists, and the arteries even of the temples scarcely perceptible; I instantly removed the dressings, cut the four lower or pubic stitches, separated the lips of the wound, removed several coagula of blood, traced the ligature to its source, found it firm and fast, and saw no oozing vessel, after repeated sponging: I therefore desisted, renewed the stitches and adhesive straps, and bound all up again. I appointed assistants to sit by her, with injunctions to examine the wound frequently, and to watch the pulse; the hands and feet were clothed with worsted stockings, and hot dressing irons applied, being previously surrounded with flannel. She remained low and exhausted, vomiting from time to time during the remainder of the evening, but began to gain strength about midnight, and turned on her right side, when she was relieved from the vomiting, and enjoyed some sleep.

"On Monday morning, she was very restless, complaining of pain in the lumbar and sacral regions, and feeling very thirsty; her pulse was so small and quick as hardly to be counted; her skin soft, and becoming warmer; her tongue a little furred and parched; there was no appearance of hemorrhage, but the binder and dressings were tinged with serous exudation coloured with blood, apparently the dropsical fluid. The urine was drawn off by the catheter. Low diet and rest enjoined. At mid-day the symptoms were improving, the pulse becoming fuller; but she felt considerable pain from the flatus in the bowels, which was not increased on full inspiration, or felt on pressure. Ordered essence of peppermint. About five in the afternoon, the pulse could be counted at the wrist, and beat 120 pulsations. In the evening she became very fatigued and thirsty, and complained more of the pain from flatus, the pain darting throughout the abdomen; but she had no pain on pressure, or taking a full inspiration. The skin was warmer, but the left arm and leg felt still cool; the pulse 127, and small; the tongue furred and parched. Urine drawn off, and the same injunctions repeated, with the administration of the peppermint. At midnight these flatulent pains were excruciating, and were relieved by the insertion of a glyster-pipe into the rectum, which allowed the escape of flatus. The other symptoms and appearances were nearly the same as in the evening. From this period, she slept at intervals during the morning, but, when awake, was excessively thirsty, which, when relieved with drink, immediately produced vomiting. She was less tormented with flatus, having been able to expel it from time to time; and she made urine naturally about six o'clock, to the quantity of ten ounces, which was very high coloured. The pulse varied from 120 to 136 during the night; and the bloody serous discharge continued to moisten the binder and other dressings.

"Tuesday morning, 1st March.—After passing a very restless night, she complained of great pain of the abdomen, in consequence of flatus, on the expulsion of which she was immediately relieved; pain was not felt on pressure, or increased by drawing a full inspiration: she complained also of excessive thirst, which, when quenched with water, plain or toast, was generally rejected. Skin comfortably warm; pulse 130, soft, and not very weak; tongue furred. In the course of the forenoon, she had a severe fit of coughing, which produced great pain in the abdomen, and caused a bloody discharge from the wound; but on examination, it was found to be deep coloured serum: it produced no faintness, or any difference in the pulse. Slept at intervals during the day, and lay tolerably quiet.

"Wednesday.—Had passed a good night, and seemed much better. Pulse 130; skin warm and soft; tongue still furred. Allowed tea, coffee, gruels, and panada.

"Thursday.—Slept soundly at intervals, and the symptoms much alleviated; was, however, occasionally very restless during the night; pulse 126, fuller and stronger; skin warm and soft; tongue cleaner. The serous discharge continued profuse. On looking at the upper part of the wound, it appeared united, and no suppuration; all was therefore left alone.

"Friday, 4th March.—Had passed a good night, and in the morning had a laxative enema administered, which operated well, and gave her great relief. Felt easy, and much less thirst. Skin warm and soft; pulse 126, stronger, fuller and softer; tongue cleaner. The wound was dressed, by changing the adhesive straps and lint: it looked extremely well, being united from one end to the other, with no inflammatory appearance; and the abdomen was soft and flaccid. She found, whenever she turned on her back, however favourable this position was to the adhesion of the wound, that she instantly vomited; she was therefore allowed to remain entirely on her side, and the right she found to be the easier.

"Saturday.—Had a good night's rest. Pulse 120, and the other symptoms as yesterday. The serous discharge much diminished, and the wound looked well. Ordered half an ounce of castor oil.

"Sunday.—Physic operated gently, producing two copious evacuations. Passed a good night, and felt much better. Symptoms and wound as on the preceding day." 13.

Having passed through the first week, we need not detail the symptoms any farther. The last report is dated 9th of May, at which time the ligature had not come away. She had been daily mending for some time previously, and was able every day to get out of bed and walk about the house.

*Case 2.* Isabella C———, aged 25 years, of spare habit and sallow complexion, was first seen by Mr. Lizars on the 13th March, labouring under cholera, and so weak that he scarcely expected her to recover. On feeling the abdomen, a large tumour was observed presenting the appearance of a sixth or seventh month utero-gestation. In a few days she had so far recovered as to be able to sit up, and was very cheerful. She earnestly requested that some operation might be performed for her relief, as she had become a burthen to herself and family. In consultation with Dr. Campbell and Mr. Christie, our author determined to operate, on Tuesday 22d March. The history of her case, as given by herself, is shortly as follows: while a servant in the north, she was one night, in January 1824, awoke with a violent pain in her right side, which continued severe till the following evening, notwithstanding laudanum internally and externally applied. In the following March she was seized in a similar manner, after which she felt her belly somewhat swollen. She had a third attack in May, and a fourth in July; after the last of which a tumour was distinctly felt in the abdomen. On consulting several gentlemen in Edinburgh she was told that she was pregnant. She continued as a cook in one of the hotels till November 1824, generally attacked once a month in the manner described. The tumour daily increased, but she remarked that it was commonly smaller in the mornings than in the evenings. Excepting during these attacks, her appetite was good, and her spirits cheerful, in spite of all the taunts of professional and unprofessional men. In this way she went on till the 13th March 1825, the time at which Mr. Lizars first saw her.

*Operation, 22d March,* 1825. The room being heated to 75°, an incision was made from the sternum to the symphysis pubis, first through the integuments, and next through the muscles and peritoneum. Mr. Lizars then began to insulate the tumour, which was found adhering so strongly to the parietes of the abdomen, to the colon, and to the brim of the pelvis, that he despaired of being able to detach it. By dissecting, however, at one time, and tearing cautiously with the fingers at another, he succeeded in insulating a large mass of a dark brown colour, weighing upwards of seven pounds, and to his joy, having a pedicle only the thickness of the little finger, and between one and two inches in length. Giving this enormous mass to his assistant, Mr. Macrae, he passed a ligature round its root, and tied it firmly, then cut it across close to the tumour, securing some open-mouthed vessels of the pedicle. During this time,

Dr. Poole kept the omentum and intestines enveloped in a warm wet towel. He now stitched up the wound, applied straps of adhesive plaister and other proper dressings, with a shawl over all. The operation was performed a little after one o'clock. At 2, the patient was extremely restless, with great pain in the wound, and pulse at 80, full and strong. At 3, same state, pulse 90 and full. At 4, the pain was abated, but the pulse had risen to 104, still very full and strong. At 5, the pulse 108, complained only of pain in the wound, and felt none on pressure of the abdomen. At 9 P. M. complained of pain in the bowels, which was increased by drink. Between this and 5 in the morning, she enjoyed some sleep, and the pulse fell to 70 in the minute. At 9 in the morning, the pulse had got up to 100 and full. At 10 A. M. the pulse was 104—bled to 14 ounces, which produced syncope. The pulse fell to 84. At 2 P. M. she had had some sleep. She is now teased with eructations and vomiting, with considerable pain—pulse 124, regular and strong. Twelve ounces more of blood abstracted —which produced syncope and some relief. At 8 P. M. the pulse 140, but she feels better, and has had some sleep. She expired at 7 o'clock the next evening.

"*Dissection.* On cutting the stitches of the wound, it was found adhering in some points, particularly at the upper or sternal part; at the lower part, a little serum and purulent matter issued out; and, on reflecting back the parietes of the abdomen, the omentum and intestines were found adhering to the neighbourhood of the wound. The peritoneum investing the parietes, which adhered to the tumour, and also those portions of this membrane investing the colon and small intestines which adhered to the tumour, were of a bluish black appearance, and tore with ease under the fingers, being evidently gangrenous. Small patches of inflammation were observable in other parts of the intestines, particularly the colon. In the pelvic cavity, there were a few ounces of serous fluid, with flakes of coagulable lymph floating in it. The pedicle was then seen to be the broad ligament of the uterus, and the uterus itself was found perfectly healthy; the other ovarium was of its natural size, had a coating of coagulable lymph, but was tolerably firm when bisected; the fallopian tube was turgid and red in the colour." 18.

*Case* 3. Magdalene B——, an unmarried woman, aged 34, observed, about six years previous to report, a tumour, the size of a hen's egg, in the right hypogastric and iliac region, without pain. Six months afterwards she received a fall, and then the pain became very severe, and the tumour rapidly increased in size, so as to compel her to go into the Infirmary of Edinburgh. From this she was dismissed, after some time, and was under the care of private practitioners. She had undergone a course of mercury, and was so far relieved as to be able to return to service, where she remained about half a year. The tumour, however, increased rapidly in size, accompanied by occasional suppression of urine, and she was forced to leave her place.

"The first time I saw her, the abdomen was fully larger than that of a woman in the ninth month of gestation, and felt hard and firm, so that I considered the tumour, from its immobility, to be strongly adhering to the parietes of the abdomen; and hence that it was an unfavourable case for operation. Her health, however, was good, she had considerable muscular strength, had a healthy countenance, a good pulse, regular bowels, but the catamenia stopt; and having entreated earnestly that something might be done, as she became less able to assist herself or her family, the 24th of April was fixed for the day of operation. On the day preceding she took a cathartic.

"The day was remarkably cold for the season, for although a large fire had been put on by seven in the morning, the thermometer had arisen only to 66° by one o'clock. When the heat of the room had arisen to 70° of Fahrenheit, which was between one and two o'clock, a longitudinal incision was made through the integuments, from the sternum to the pubes: at the sternal extremity the peritoneum was wounded, and one finger of the left hand was here introduced, then another, and the peritoneum laid open to the pubes: the same was then done upwards to the sternum, when a multiplicity of convoluted vessels presented themselves of various magnitude, from the thickness of a finger to that of a crow's quill. At first I thought them the intestines, for they appeared extremely fleshy; then I imagined them the blood-vessels of a placenta, which they still more resembled; indeed, such was their resemblance to the vessels of that organ, that the same idea struck one and all the gentlemen present. On minute examination, however, they were found to be the blood-vessels of the omentum majus, enormously enlarged, running on the surface, and into the substance of the tumour, which appeared an enlarged ovarium. Finding that it was impracticable either to dissect these vessels from the surface of the tumour, or to secure them, in consequence of their great number, I abandoned the idea of extirpating the mass, in which decision I was supported by the gentlemen present; I therefore punctured with a large trocar and canula the centre of the tumour, but nothing flowed; I next made a small but deep incision with a scalpel, when the tumour appeared solid and cartilaginous, and a vessel bled a little: I lastly punctured the lower part of the tumour, being anxious to reduce its bulk, but only pure blood flowed. The lips of the wound were now approximated and stitched; adhesive straps applied, compresses of lint and linen, with a shawl as a binder, and the patient carried to bed. This operation was performed in the presence of James Dease, Esq. Surgeon to the forces; Drs. Poole, Campbell, and Millar; Messrs James Scott, George White, and many other surgeons and students." 20.

The details of the after-treatment are too tedious to notice here; but it may be sufficient to say that the patient recovered from the operation, the original disease remaining the same.

We have now put our readers in possession of the main facts of the present publication. Of the three cases now detailed, and the one formerly stated, in all, four in number, we find that in the first case, no tumour could be discovered—in the second, the operation was successful, up to the date of report—in the third case, death was the consequence—and in the fourth, the operation could not be continued, after laying open the abdomen. These results are not very favourable to the repetition of such operations, although the successful termination of one of them proves the possibility of the operation under particular circumstances. These circumstances are what were witnessed in case No. 1:—namely, the completely detached state of the tumour, growing from a small pedicle. Such favourable circumstances are not likely often to exist—for we find the adhesion of abdominal tumours to the neighbouring parts an almost general occurrence. The operations detailed do great credit to the intrepidity of the operator, and he deserves our meed of praise. Still we are not altered in our opinion respecting the policy, not to say the propriety of similar operations in future. The plates, as might be expected, are executed with great ability.

